# Biological impacts on the lungs in rats internally exposed to radioactive ^56^MnO_2_ particle

**DOI:** 10.1038/s41598-021-90443-9

**Published:** 2021-05-26

**Authors:** Nariaki Fujimoto, Bakhyt Ruslanova, Zhaslan Abishev, Nailya Chaizhunussova, Dariya Shabdarbayeva, Gaukhar Amantayeva, Rakhimzhanova Farida, Marat Sandybayev, Kasuke Nagano, Kassym Zhumadilov, Andrey Kaprin, Sergey Ivanov, Valeriy Stepanenko, Masaharu Hoshi

**Affiliations:** 1 Research Institute for Radiation Biology and Medicine, Hiroshima University, Hiroshima, Japan; 2 Semey Medical University, Semey, Kazakhstan; 3 Center of Nuclear Medicine and Oncology of Semey, Semey, Kazakhstan; 4 Nagano Toxicologic-Pathology Consulting, Kanagawa, Japan; 5 L.N. Gumilyov Eurasian National University, Nur-Sultan, Kazakhstan; 6 National Medical Research Center of Radiology, Ministry of Health of Russian Federation, Obninsk, Russia; 7 The Center for Peace, Hiroshima University, Hiroshima, Japan

**Keywords:** Biochemistry, Environmental sciences, Health care, Risk factors

## Abstract

To understand the radiation effects of the atomic bombing of Hiroshima and Nagasaki among the survivors, radiation from neutron-induced radioisotopes in soil should be considered in addition to the initial radiation directly received from the bombs. ^56^Mn, which emits both β particles and γ-rays, is one of the dominant radioisotopes created in soil by neutrons from the bomb. Thus we investigated the biological effects of internal exposure to ^56^MnO_2_ particle in the lung of male Wistar rats comparing to the effects of external ^60^Co-γ irradiation. Absorbed doses of internal irradiation of lungs were between 25 and 65 mGy in ^56^MnO_2_-exposed animals, while the whole body doses were between 41 and 100 mGy. Animals were examined on days 3 and 61 after the exposure. There were no remarkable pathological changes related to ^56^MnO_2_ particle exposure. However, mRNA and protein expressions of aquaporin 5 increased significantly in the lung tissue on day 3 postexposure in ^56^MnO_2_ groups (by 1.6 and 2.9 times, respectively, in the highest dose group). Smad7 mRNA expression was also significantly elevated by 30% in the highest dose group of ^56^MnO_2_. Our data demonstrated that internal exposure to ^56^MnO_2_ induced significant biological responses including gene expression changes in the lungs, while external ^60^Co-γ irradiation of 2 Gy did not show any changes.

## Introduction

On the atomic bombing in Hiroshima and Nagasaki, Japan, initial radiation directly from the explosions caused major biological effects. However, residual radiation may have played a role because the people who moved to these cities soon after the detonations not received any direct radiation but exposed to residual-radioactive dust were reported to suffer from various acute radiation syndrome^1^. The radioisotopes produced in soil by the neutron from the atomic bomb included ^24^Na, ^42^K, and ^56^Mn^2^. Considering human exposures, ^56^Mn, which was existed as insoluble ^56^MnO_2_ particles, was one of the most important sources of residual radiation. However, there had been no studies examining the effects of internal exposure to ^56^Mn before. Then we initiated to investigate the biological effects of this residual radioisotope by exposing laboratory rats to ^56^MnO_2_ particles and reported possible pathological changes in the lung^3^. Although our previous report consisting of two separate exposure experiments pioneered the investigations of the biological effects of ^56^Mn particles in animals, the obtained biological data were limited. In the first exposure experiment, only one rat was examined at each time point after exposure to ^56^Mn particles with a single dose of radiation. In the second exposure, the radiation dose did not reach the level as planned, so the pathological results were less clear. Therefore, a new study to investigate the effects of ^56^Mn on the lung had been awaited to validate our previous findings and to obtain more detailed data (Supplementary Information).

In the present study, we exposed the necessary numbers of male Wistar rats to three different doses of radioactive ^56^MnO_2_ powder, or stable MnO_2_ powder to further examine our previous findings. To compare the effects of internal irradiation by ^56^MnO_2_ with the external irradiation’s effects, a group exposing to ^60^Co-γ at 2 Gy, which is substantially higher than the predicted whole body doses by ^56^MnO_2_, was also prepared. The dosimetry data indicated that the organ distribution of internal radiation in the present study was similar to the previous results^4^, suggesting good reproducibility in the ^56^MnO_2_ exposure process^5^. In general, thoracic external radiation exposure at high doses (> 8 Gy) induces radiation pneumonitis which eventually leads to pulmonary fibrosis in laboratory animals as well as in humans^6–9^. Investigations of developing mechanisms of pulmonary fibrosis have suggested the involvement of transforming growth factor-β (TGF-β) and the related genes, including the Smad genes^10–12^. Aquaporins (AQPs) are water channel molecules that control water transfer across cellular membranes. In the lung, AQP1 is located in microvascular endothelia, while AQP 4 is in airway epithelia and AQP 5 is in type I alveolar epithelial cells, all of which are involved in the transport of liquid to maintain a normal physiological status of the lung^13^. Expressions of AQPs were altered in the pathological conditions: AQP1 expression was decreased in the lung with acute hypoxic lung injury, while adenovirus infection decreased both AQP1 and AQP4^14^. Thoracic irradiation could also affect the pulmonary expression of AQP1 and AQP5 in rats^15^. Therefore, to identify the radiation effect on the lung, we determined the lung injury related gene expressions including TGF-β, Smad2, Smad7, collagen I, elastin, and AQPs 1, 4 and 5, in addition to the histological analysis of the lung. The animals were examined on days 3 and 61 postexposure because our previous study indicated that pathological changes occurred in the lung on day 3 and were recovered by day 60^3^.

## Results

### Estimated doses of irradiation

The estimated accumulated doses of internal irradiation from ^56^MnO_2_ in each organ were previously described^5^. The absorbed doses of the internal irradiation of the lungs were 25 ± 3 (Mn56x1 group), 48 ± 9.0 (Mn56x2 group), and 65 ± 13 (Mn56x3 group), while the doses of internal irradiation of the whole body were 41 ± 8, 91 ± 3, and 100 ± 10 mGy, respectively. The external irradiation dose from ^60^Co-γ exposure was 2.0 ± 0.08 Gy.

### Body and lung weights

Body and lung weights on days 3 and 61 postexposure are summarized in Table 1. There were no significant differences in lung weight on either day.

**Table 1:** Body and lung weights in rats exposed to ^56^MnO_2_, ^60^Co γ-rays, and cold MnO_2_.

Groups	Body weight (g)	Lung (g)	Lung (relative) (g/kg bw)
**Day 3**			
Control	248 ± 16	1.50 ± 0.15	6.0 ± 0.5
Cold Mn	235 ± 14	1.46 ± 0.12	6.3 ± 0.6
Mn56x1	235 ± 11	1.29 ± 0.07	5.6 ± 0.4
Mn56x2	245 ± 16	1.52 ± 0.12	6.3 ± 0.5
Mn56x3	237 ± 12	1.35 ± 0.08	5.7 ± 0.2
Co-60	234 ± 14	1.29 ± 0.09	5.6 ± 0.4
**Day 61**			
Control	330 ± 17	2.10 ± 0.13	6.5 ± 0.5
Cold Mn	337 ± 19	1.78 ± 0.05	5.3 ± 0.3
Mn56x1	371 ± 21	1.82 ± 0.13	4.9 ± 0.2
Mn56x2	337 ± 17	1.99 ± 0.13	6.0 ± 0.5
Mn56x3	353 ± 17	1.84 ± 0.11	5.2 ± 0.3
Co-60	328 ± 23	2.04 ± 0.12	6.4 ± 0.5

Each value shows mean ± SEM (n = 6 or 7, each group).

### Histology of the lung

**Figure 1:**
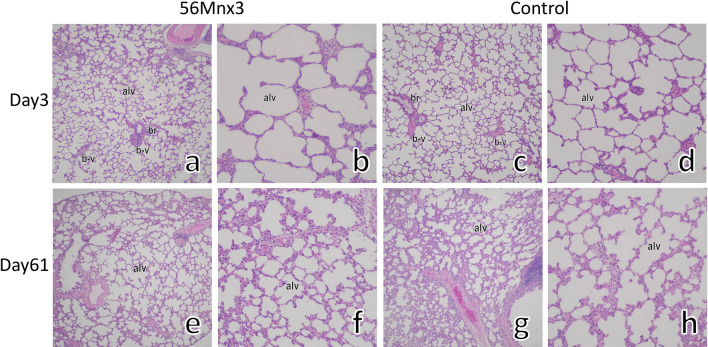
The rats' lungs on days 3 (**a**–**d**) and 61 (**e**–**h**) after ^56^MnO_2_ exposure and the control (HE staining). There were no significant histological alternations in the lungs among Mn56x3 (**a**,**b**,**e**,**f**) and the control (**c**,**d**,**g**,**h**) groups. The structure of alveoli (alv) was normal with bronchiole (br) and blood vessels (b-v) in the Mn56x3 group. There were no signs of thickening of the alveolus wall even on day 61 postexposure (**f**) compared to the control (**h**). Original magnification × 40 (**a**,**c**,**e**,**g**), or × 100 (**b**,**d**,**f**,**h**).

**Figure 2:**
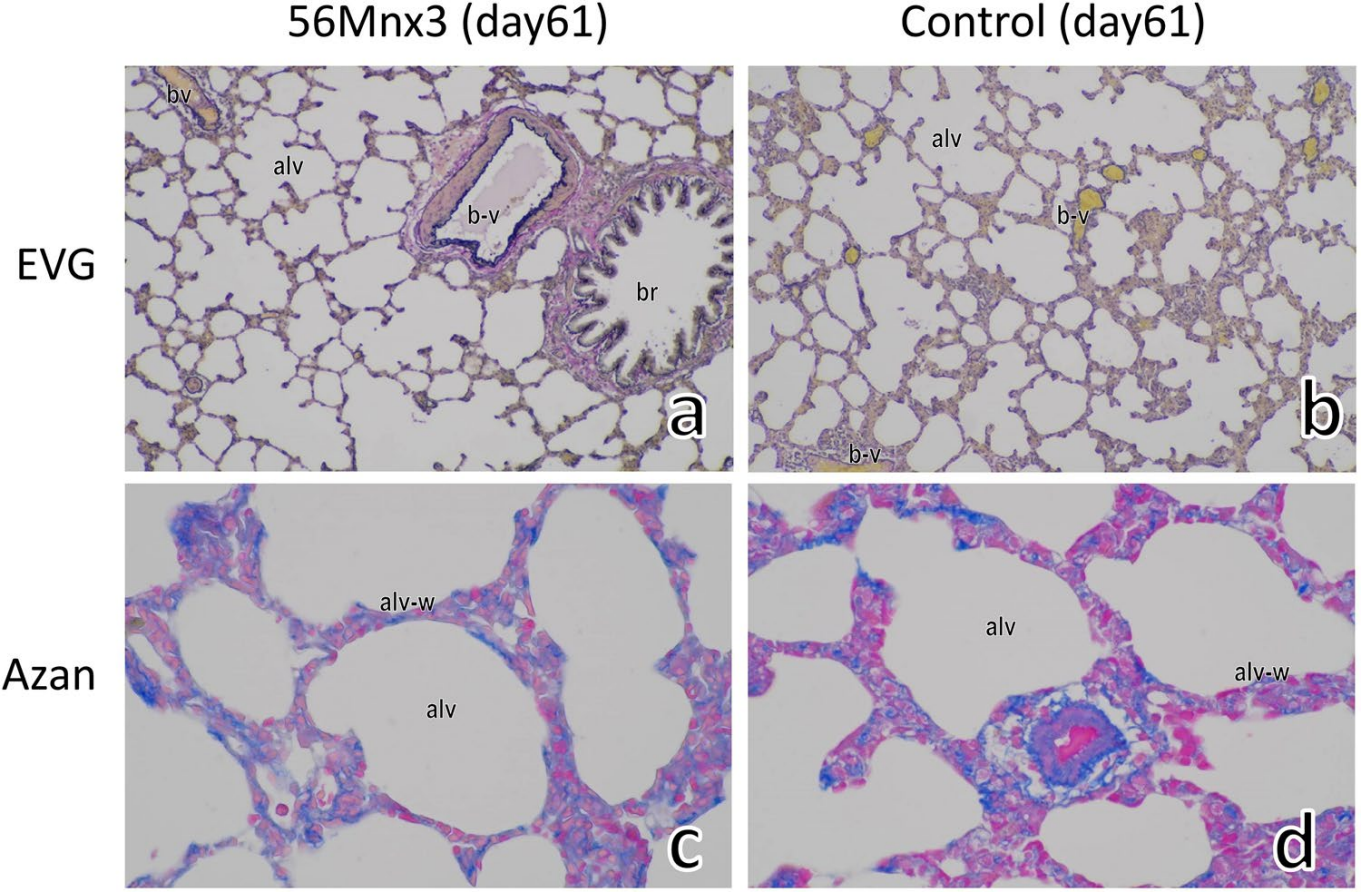
The lung of rats on day 61 after ^56^MnO_2_ exposure (**a**,**c**) and the control (**b**,**d**). EVG staining indicated no significant changes in elastin (dark blue or black color) in the alveoli (alv) by ^56^Mn exposure (**a**). Azan staining showed no signs of increased collagen deposition (blue color) in the alveolus wall (alv-w) in the ^56^Mnx3 group (**c**). Original magnification, × 100 (**a**,**b**), or × 400 (**c**,**d**).

Representative histology of the lung with hematoxylin and eosin (HE) staining on days 3 and 61 postexposure in Mn56x3 and the control are shown in Fig. 1. There were no remarkable changes in the lungs in any group. The structure of the alveoli was normal with no signs of thickening of the alveolus wall. Figure 2 showed representative Elastica van Gieson (EVG) and Azan stained histological sections in Mn56x3 and control. EVG staining did not indicate any deposition of elastin on day 61 postexposure. There were no signs of an increase in collagen in the alveolus wall in Azan staining either.

### Effects on mRNA expression levels of Col-I, elastin, TGF-β, Smad2, and Smad7

**Figure 3:**
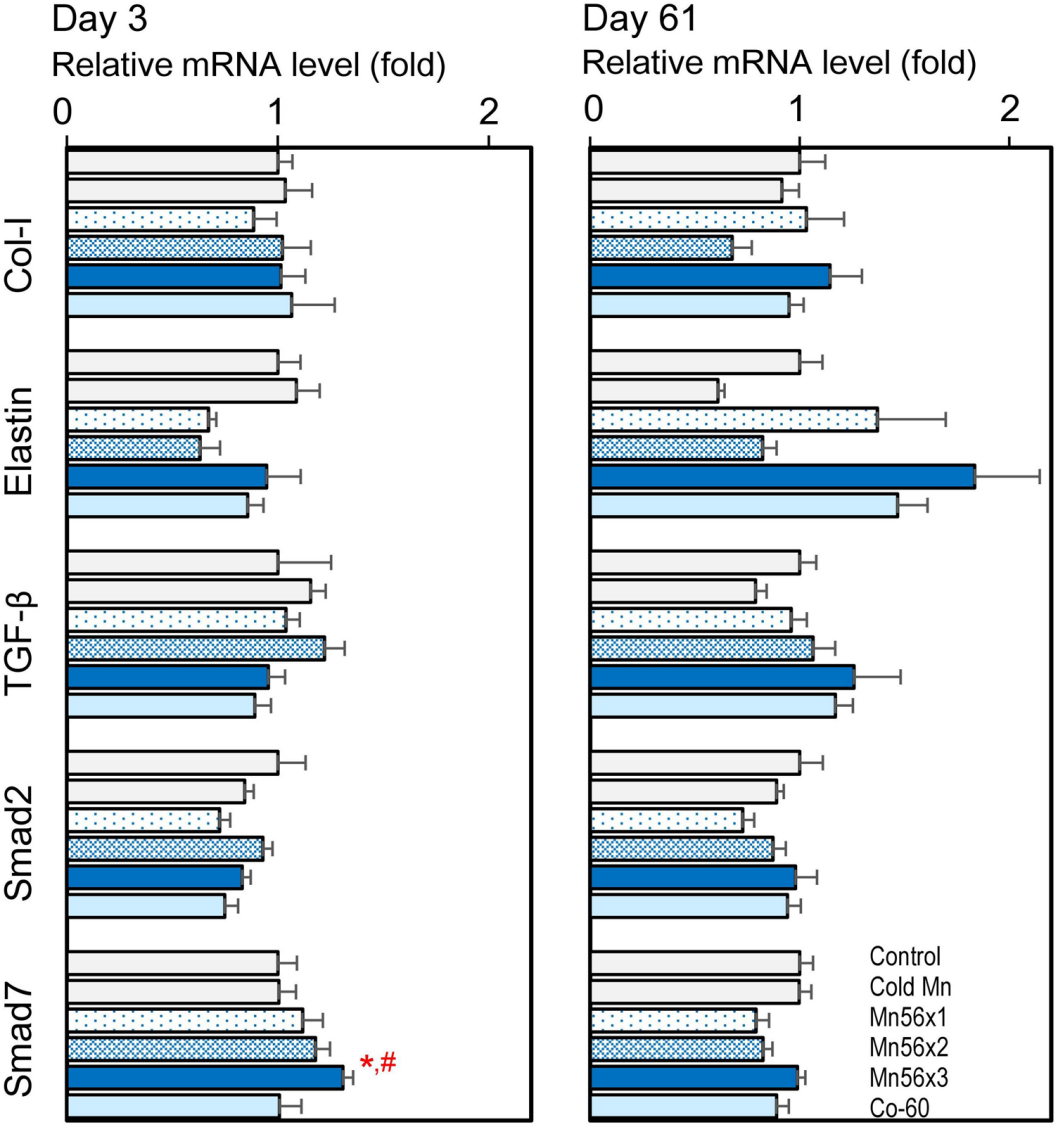
Relative mRNA expression levels of collagen-I, elastin, TGF-β, Smad2 and Smad7 genes in the lungs of rats on day 3 (left) and day 61 (right) after the exposure to ^56^MnO_2_ powder (Mn56x1, Mn56x2, Mn56x3), Cold MnO_2_ powder (Cold Mn) or ^60^Co-γ exposure (Co-60). *p < 0.05 vs. control; ^#^p < 0.05 vs cold Mn.

Figure 3 shows the expression levels of radiation-induced fibrosis-related genes, Col-I, Elastin, TGF-β, Smad2, and Smad7. In the Mn56x3 group, the Smad7 mRNA levels were significantly elevated on postexposure days 3. There were no changes in mRNA level otherwise.

### Effects on mRNA expression levels of AQP1, AQP4, and AQP5

**Figure 4:**
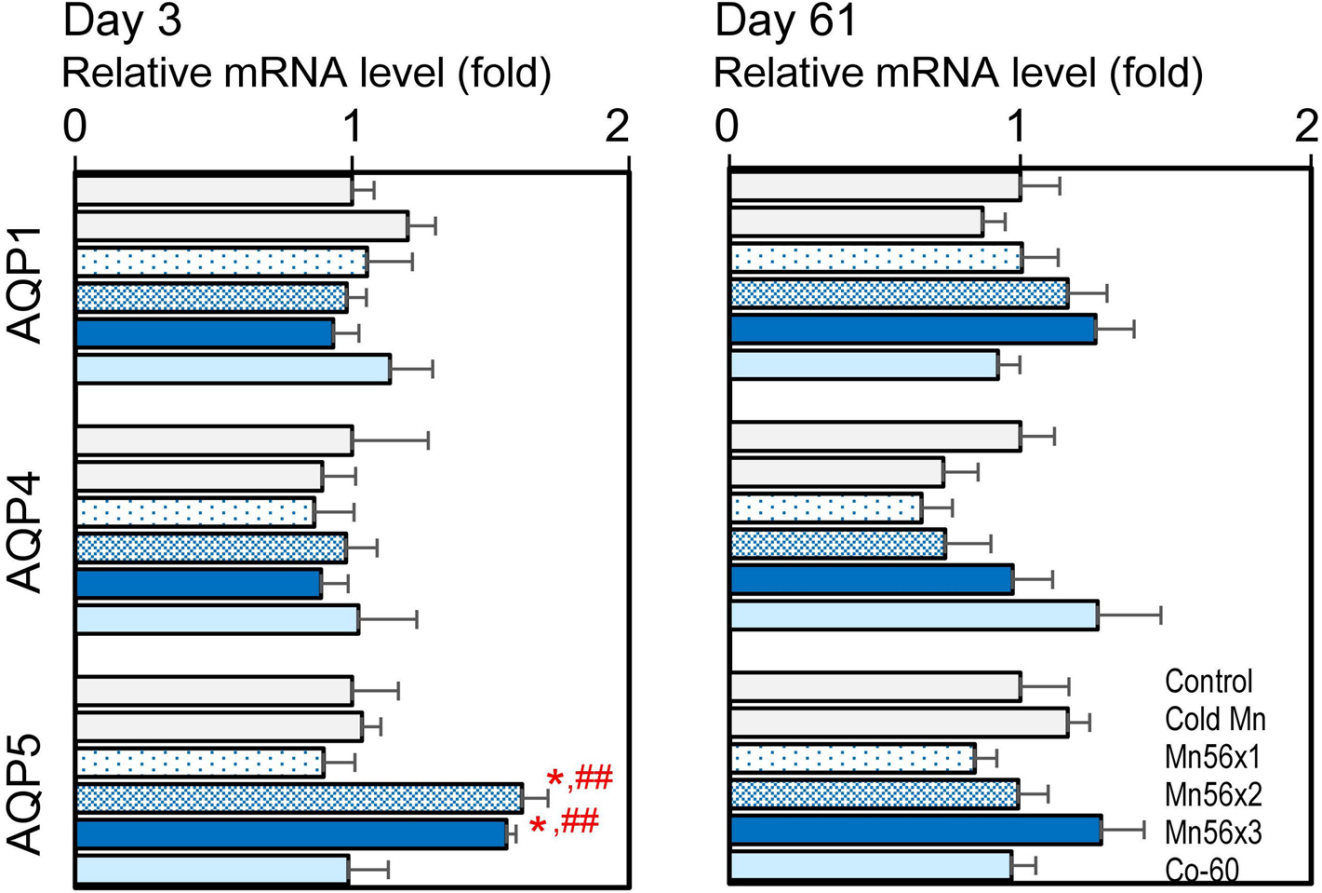
Relative mRNA expression levels of AQP1, AQP4, and AQP5 genes in the lungs of rats on day 3 (left) and day 61 (right) after the exposure to ^56^MnO_2_ powder (Mn56x1, Mn56x2, Mn56x3), Cold MnO_2_ powder (Cold Mn) or ^60^Co-γ exposure (Co-60). *p < 0.05 vs. control; ^##^p < 0.01 vs cold Mn.

Figure 4 presents the mRNA expression of AQPs 1, 4, and 5 in each group. On postexposure day 3, AQP5 mRNA expression significantly increased in the Mn56 groups, while AQP1 and AQ4 mRNA levels were not affected. There were no changes on postexposure day 61.

### AQP5 protein expression

**Figure 5:**
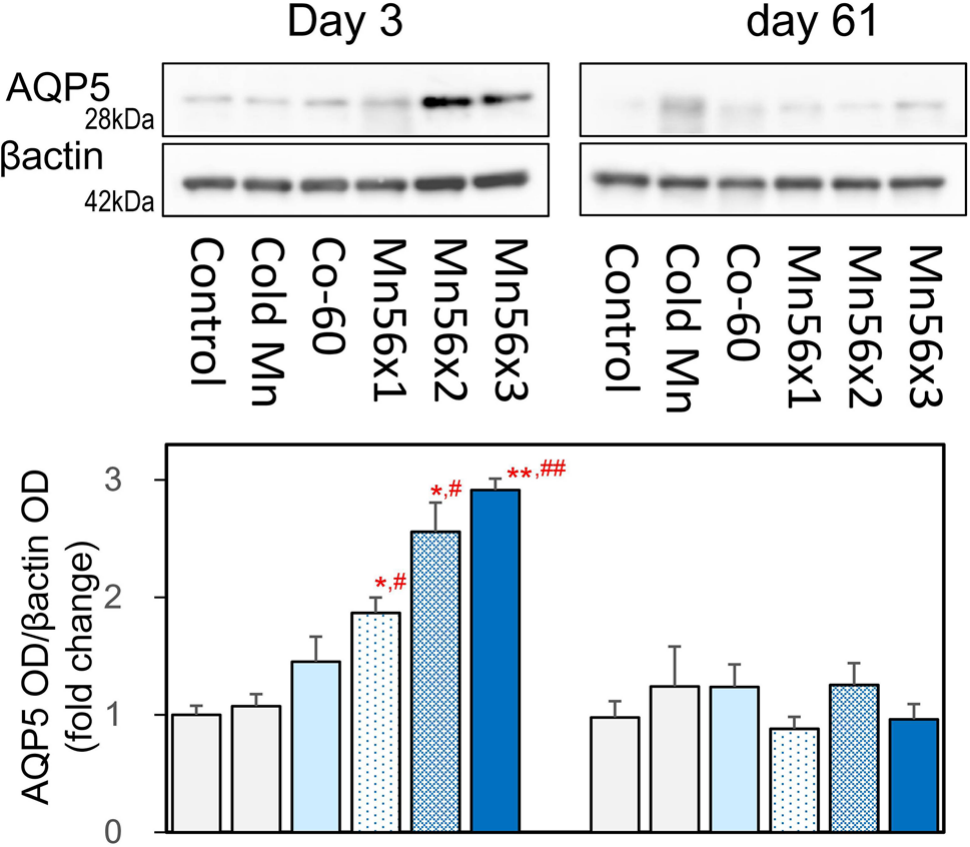
Western blot analysis and densitometry for AQP5 in the lung on postexposure days 3 and 61. β-Actin was presented as an internal control. Lysates extracted from the lung were examined. *p < 0.05, **p < 0.01 vs. control; ^#^p < 0.05, ^##^p < 0.01 vs cold Mn.

Figure 5 showed the AQP5 protein expression in the lungs. The western blotting analysis demonstrated that AQP5 expression was significantly increased in the protein levels in both Mn56x2 and Mn56x3 groups on postexposure day 3.

## Discussion

Previously we examined male Wistar rats exposed to ^56^MnO_2_ powder to understand the biological effects of the possible residual radioactive particles from atomic bombing^3^. Although we reported pathological changes in the lung, it was based on the observation of one animal each day 3, 14, or 60 after the exposure. Thus, in the present study, we conducted a new experiment with a substantial number of rats exposed to radioactive ^56^MnO_2_ particles at three different doses to further examine the effects on the lungs by pathological analysis and determination of the gene and protein expression changes. It was difficult to obtain higher radiation doses of ^56^Mn than the previous levels due to the technical limitation of our facility. No pathological changes were found even in the Mn56x3 group. However, the expression in AQP5 increased both mRNA and protein levels on postexposure day 3 in the Mn56x2 and Mn56x3 groups. The Smad7 mRNA expression was also elevated in the Mn56x3 group. However, there were no such changes in the Co-60 group. These data suggest that the internal exposure to ^56^MnO_2_ powder induced significant biological responses in the lung tissue than external ^60^Co-γ irradiation of 2 Gy although the effects were not pathologically apparent.

It is important to include the effects of residual radiation to understand the radiation effects among the survivors of the atomic bombing of Hiroshima and Nagasaki. Since neutron-activated radioisotopes were present in dust after the bombings, people might have inhaled these radioactive materials and been internally exposed to the radiation. Individuals, who returned early to Hiroshima and Nagasaki after the atomic bombing, were reported to suffer from the symptoms of acute radiation effects^1^. Since there are no data available regarding the absorbed doses in the lung of atomic bomb survivors by these residual radioactive dust, it is not clear whether the radiation doses in the lung found in the present study represent the reality of atomic bomb survivors. However, our data demonstrated the possibility that internal exposure to residual radiation from ^56^Mn had played a role in developing radiation-induced disorders among the atomic bomb survivors.

High doses of external radiation exposure to the lungs can induce radiation pneumonitis followed by fibrosis in laboratory animals and humans^7,16^. Radiation-induced lung injury is a major dose-limiting factor in thoracic radiotherapy and has been extensively investigated^17^. TGF-β plays an essential role in the development of radiation-induced pulmonary lesions. Serum TGF-β could be used as a predictor of radiation pneumonitis in humans^18^. Mice exposed to thoracic irradiation also showed an acute and long-lasting increase in pulmonary TGF-β expression^19^. Although TGF-β is involved in physiological wound healing and possible subsequent fibrosis, this process often leads to chronic pathological results. TGF-β binds to its cell surface serine/threonine kinase receptors, which phosphate the Smad proteins to initiate the transcription of the genes related to diverse biological processes, including fibrosis. Irradiation may change the expression levels of Smad genes, as it was reported that Smad7 gene expression was increased while Smad2 gene expression was decreased in the lung tissues of Wistar rats irradiated with thoracic X-rays at 20 Gy^12^. In a study with mice exposed to thoracic X-rays at 12 Gy, the Smad signaling was altered on days 2 and 17 postexposure^20^. The present study determined the mRNA levels of TGF-β, Smad2, and Smad7 genes to examine the possible effects of exposure to ^56^MnO_2_. External ^60^Co exposure at 2 Gy did not cause any changes in these gene expressions. Interestingly, however, Smad7 mRNA levels were significantly increased in the Mn56x3 group on postexposure day 3, while no differences were noted otherwise. This temporal increase in Smad7 may relate to the healing processes of the lung tissue damages, which were not pathologically apparent. In the development of lung pneumonitis and fibrosis after radiation or chemical exposures, both collagen and elastin contents gradually increase in the lung tissue as early as two weeks after the exposure^21,22^. In the present study, however, there were no significant changes in expressions in either collagen or elastin, which suggested the ^56^Mn exposure may not lead to fibrotic disorders. Aside from ^56^Mn, the effects of radionuclides on the lung have been investigated extensively, although most of these early studies have focused on the development of lung cancer^6^. Some of these studies demonstrated that rats exposed to the α-emitting particles such as ^239^PuO_2_ or β-emitting particles including ^147^Pm indeed induced pneumonitis at the high doses^23,24^. Since our data showed that the change in Smad7 expression occurred even at the low internal dose of 64 mGy, it would be interesting to investigate the gene expression profiles in the lung exposure to the other radionuclides as well. Other gene expression changes reported in the irradiated lung representing biological responses were AQPs, a water-selective channel protein family. AQPs are ubiquitously expressed throughout the body and facilitate plasma membrane water permeability and fluid movement^25^. In the lung, AQP1 is expressed in the capillary endothelium and AQPs 4 and 5 are in the epithelium, which plays important roles in the lungs' physiology and pathology^26^. In experimentally induced pulmonary edema in rats, AQP5 expression was found to increase in both mRNA and protein levels, which is probably a physiological response to prevent the lung from edema^27^. For radiation effect studies in rats X-irradiated thoracically, it was reported that AQP5 mRNA level was increased while AQP1 mRNA were decreased in 7 days after the exposures which also suggested a protective role against the radiation-induced inflammation and edema^15^. The present study showed an upregulation of AQP5 mRNA and protein in Mn56x2 and Mn56x3 groups on postexposure day 3, which could be physiological responses and may indicate minor lung injuries caused by ^56^MnO_2_ exposure, although the histological examination did not indicate any damages. There were no changes in AQP5 or the other gene expression in the Co-60 group, which indicated that internal exposure to ^56^Mn had higher biological impacts on the lung tissue than the external 2 Gy of γ-irradiation.

The present study did not identify any pathological effects of ^56^MnO_2_ exposure on the lung using a significant number of rats. The previous pathological findings were not reproducible. However, it is worth noting that 6-month-old male Wistar rats were used in the previous study, while 10-week-old Wistar rats were used in the present study. The age factor may have caused the discrepancy in the results between the two separate experiments. Mn is known as a neurotoxic chemical at high doses^28^. In the present study, animals were exposed to MnO_2_ for only 1 h, which probably did not produce any chemical toxicity of Mn. In fact, there were no significant differences in any parameters examined between cold-Mn group and the control (Table 1, and Figs. 3, 4, 5). To investigate the radiation-induced lung injuries, mouse models of C57BL and C3H strains were well established^29,30^. These mouse models would be useful to understand further the effects of ^56^MnO_2_ particles.

In conclusion, ^56^MnO_2_ exposure at internal doses of 25–64 mGy did not induce any significant pathological changes in the lungs. However, it caused significant impacts on pulmonary gene and protein expressions, including upregulation of AQP5, and Smad7 expressions, which were not observed in external γ-irradiation of 2 Gy, indicating very high biological impacts of internal radiation from ^56^MnO_2_ particles and suggesting the possible roles in radiation effects among atomic bomb survivors.

## Methods

### Animals

The animal experiment design was previously described^5,31^. Specific pathogen-free male Wistar rats (10 weeks old) were purchased from the Kazakh Scientific Center of Quarantine and Zoonotic Diseases, Almaty, Kazakhstan. The animals had free access to a basal diet and tap water. The animal facility conditions were maintained as follows: a room temperature of 19–22 °C, a relative humidity of 30–70%, and a 12-h light cycle. Rats were randomly divided into six groups: Mn56x1 (*n* = 17), Mn56x2 (*n* = 17), Mn56x3 (*n* = 17), Co-60 (*n* = 14), cold Mn (*n* = 14), and control (*n* = 14). The Mn56x1, Mn56x2, and Mn56x3 groups were exposed to ^56^MnO_2_ powder (100 mg) at activities of 2.7 × 10^8^, 5.5 × 10^8^, and 8 × 10^8^ Bq, respectively. The cold Mn group was exposed to nonradioactive MnO_2_ powder (100 mg). Three rats from each Mn56 group were used to determine the absorbed radiation doses. The Co-60 group received 2 Gy of external ^60^Co γ-ray whole-body irradiation. From each group, seven rats underwent necropsies on postexposure days 3 and 61. The rats were sacrificed by removing whole blood under anesthesia with isoflurane (Fujifilm Wako Pure Chemical Co., Tokyo, Japan). The lung was dissected and stored in RNA Save solution (Biological Industries Ltd, Beit Alfa, Israel) for RNA extraction, and part of it was fixed in 10% formalin. The animal study was approved by the Animal Experiment Ethics Committee of Semey Medical University, Semey, Kazakhstan (document #3-30.11.2018). The study was carried out in compliance with the ARRIVE guidelines.

### Irradiation and dosimetry

Preparation and exposure of ^56^MnO_2_ powder and the internal dose estimation have been previously described^4,5^. Briefly, MnO_2_ powder (Rare Metallic Co., Tokyo, Japan; particle diameters range, 1–19 µm) was activated by neutron beam in the Baikal-1 nuclear reactor at the National Nuclear Center, Kurchatov, Kazakhstan. The obtained radioactive ^56^MnO_2_ powder was sprayed over the rats for 1 h. Then three rats per group were sacrificed for gamma spectrometry and estimated each organ's absorbed dose. All animals were brought to the ^56^Mn exposure facility. A ^60^Co radiotherapy machine, Teragam K-2 unit (UJP Praha, Praha-Zbraslav, Czech Republic) was used (2 Gy at 1.0 Gy/min) for whole-body γ-ray irradiation. A radiophotoluminescence glass dosimeter, GD-302M (Chiyoda Technol Co., Tokyo, Japan), was used to estimate ^60^Co dose.

### Pathology

Formalin-fixed tissues were embedded in paraffin. Sections of 4 µm thickness were prepared and stained with HE. Sections were stained with Azan for pulmonary interstitial collagen evaluation. EVG staining was applied for evaluating elastic fiber.

### Measurement of mRNA levels by a quantitative reverse transcription-polymerase chain reaction

The details were described previously^32^. Total RNA was prepared using Isogen II (Nippon Gene Co., Tokyo, Japan) from pieces of the lung tissue stored in the RNA Save solution. The cDNA was synthesized by incubating 3 µg of total RNA + RevaTra Ace 100 U (Toyobo Co., Osaka, Japan) + 20 pmol random hexamers/5 pmol oligo-dT(15) primers (Takara Bio Inc., Kusatsu, Japan). The quantitative polymerase chain reaction (qPCR) system, StepOnePlus (Applied Biosystems, Life Technologies, Co., Carlsbad, CA, USA), was employed for cDNA measurement with a KAPA SYBR Fast qPCR Kit (Kapa Biosystems, Inc., Woburn, MA, USA). PCR conditions have 30 s initial denaturation followed by 40 cycles of 5 s at 95 °C and 35 s at 60 °C. Specific primer sets for the lung genes are listed in Table 2. The PCR products were prepared, and the nucleotide sequences were confirmed by Fasmac Co., Ltd. (Atsugi, Japan) before analysis. The measured mRNA levels were normalized according to the levels of β-actin mRNA.

**Table 2:** Q-PCR primers.

Gene	GenBank accession#	Q-PCR primer sequences (5′→3′)	
		Forward	Reverse
Aqp1	NM_012778	CCACTGGAGAGAAACCAGACG	CTGAGCAGAAGCCCCAGTGT
Aqp4	NM_001317749	TCTGGACTCAAGCCTTCTGGAA	AGTCCAAAGCAGAGGGAGATGA
Aqp5	NM_012779	AGGCATCCTGTACTGGCTGG	GAGGAGAAGATGCAGAGGGCT
Col1a1	NM_053304	AACCTGGATGCCATCAAGGTC	GCTCTCTCCAAACCAGACATGC
Ela	BC085910	TCCTATCTACCCAGGTGGTGG	CACTTTCTCTTCCGGCCACA
TGFβ	AY550025	GCTGAACCAAGGAGACGGAAT	GAAGGGTCGGTTCATGTCATG
Smad2	AB010147	GCTCTCCGGCTGAACTGTCT	TGTGACGCATGGAAGGTCTCT
Smad7	NM_030858	TTGCTGTGAATCTTACGGGAAG	GGTTTGAGAAAATCCATCGGGT

### Western blot

Lung tissues were homogenized in RIPA lysis buffer containing phosphatase inhibitors (Santa Cruz Biotechnology Inc., Santa Cruz, CA, USA). Two milligrams of each lysate were applied to 12.5% sodium dodecyl sulfate-polyacrylamide gel electrophoresis and transferred to Hybond-P membranes (GE Healthcare Ltd, Buckinghamshire, UK). After blocking with Blocking One-P (Nacalai Tesque Inc, Kyoto, Japan), the membranes were incubated with anti-AQP5 (GTX11586, x2000, GeneTex, Inc., Irvine, CA, USA) or anti-β-actin (PM053, 1:1000, MBL Co., Nagoya, Japan). They were washed and incubated with peroxidase-conjugated anti-rabbit IgG (1:5000, MBL Co.). The protein bands were visualized with Chemi-Lumi reagents (Nacalai Tesque Inc) and image-captured with a CCD camera system, ImageQuant LAS 4000 mini (GE Healthcare Ltd).

### Statistical analysis

All values are expressed as mean ± standard error of the mean. Dunnett's test compared each treated group with the control group or the cold Mn group.

### Ethical approval

All methods were carried out in accordance with relevant guidelines and regulations.

## Supplementary Information

Supplementary Information.
