# Supplementary material for: Biological impacts on the lungs in rats internally exposed to radioactive 56MnO2 particle
Source: Sci Rep. 2021 May 26;11:11055. doi: 10.1038/s41598-021-90443-9 (PMC8155131; doi:10.1038/s41598-021-90443-9)
Supplement: Supplementary file 1 — Supplementary Information. [file 41598_2021_90443_MOESM1_ESM.pdf]

Supplemental Information

Biological impacts on the lungs in rats internally exposed to radioactive $^{56}MnO_2$ particle

Nariaki Fujimoto[1*], Bakhyt Ruslanova[2], Nailya Chaizhunussova[2], Dariya Shabdarbayeva[2], Zhaslan Abishev[2], Gaukhar Amantayeva[2], Rakhimzhanova Farida[2], Marat Sandybayev[3], Kasuke Nagano[4], Kassym Zhumadilov[5], Sergey Ivanov[6], Valeriy Stepanenko[6], Masaharu Hoshi[7]

[1]Research Institute for Radiation Biology and Medicine, Hiroshima University, Hiroshima, Japan

[2]Semey Medical University, Semey, Kazakhstan

[3]Center of Nuclear Medicine and Oncology of Semey, Semey, Kazakhstan

[4]Nagano Toxicologic-Pathology Consulting, Kanagawa, Japan

[5]L.N. Gumilyov Eurasian National University, Nur-Sultan, Kazakhstan

[6]A. Tsyb Medical Radiological Research Center, National Medical Research Center of Radiology, Ministry of Health of Russian Federation, Obninsk, Russia

[7]The Center for Peace, Hiroshima University, Hiroshima, Japan

* nfjm@hiroshima-u.ac.jp

ARRIVE

The ARRIVE Guidelines Checklist

Animal Research: Reporting In Vivo Experiments

Carol Kilkenny[1], William J Browne[2], Innes C Cuthill[3], Michael Emerson[4] and Douglas G Altman[5]

[1]The National Centre for the Replacement, Refinement and Reduction of Animals in Research, London, UK, [2]School of Veterinary Science, University of Bristol, Bristol, UK, [3]School of Biological Sciences, University of Bristol, Bristol, UK, [4]National Heart and Lung Institute, Imperial College London, UK, [5]Centre for Statistics in Medicine, University of Oxford, Oxford, UK.

	ITEM	RECOMMENDATION	Section/ Paragraph
Title	1	Provide as accurate and concise a description of the content of the article as possible.	Title
Abstract	2	Provide an accurate summary of the background, research objectives, including details of the species or strain of animal used, key methods, principal findings and conclusions of the study.	Abstract
**INTRODUCTION**			
Background	3	a. Include sufficient scientific background (including relevant references to previous work) to understand the motivation and context for the study, and explain the experimental approach and rationale.<br>b. Explain how and why the animal species and model being used can address the scientific objectives and, where appropriate, the study's relevance to human biology.	P3L2-16<br>P10L4-14
Objectives	4	Clearly describe the primary and any secondary objectives of the study, or specific hypotheses being tested.	P3L17-P4L16
**METHODS**			
Ethical statement	5	Indicate the nature of the ethical review permissions, relevant licences (e.g. Animal [Scientific Procedures] Act 1986), and national or institutional guidelines for the care and use of animals, that cover the research.	P10L17-19
Study design	6	For each experiment, give brief details of the study design including:<br>a. The number of experimental and control groups.<br>b. Any steps taken to minimise the effects of subjective bias when allocating animals to treatment (e.g. randomisation procedure) and when assessing results (e.g. if done, describe who was blinded and when).<br>c. The experimental unit (e.g. a single animal, group or cage of animals).<br>A time-line diagram or flow chart can be useful to illustrate how complex study designs were carried out.	P10L8-9<br>P10L8-9<br>P10L4-8
Experimental procedures	7	For each experiment and each experimental group, including controls, provide precise details of all procedures carried out. For example:<br>a. How (e.g. drug formulation and dose, site and route of administration, anaesthesia and analgesia used [including monitoring], surgical procedure, method of euthanasia). Provide details of any specialist equipment used, including supplier(s).<br>b. When (e.g. time of day).<br>c. Where (e.g. home cage, laboratory, water maze).<br>d. Why (e.g. rationale for choice of specific anaesthetic, route of administration, drug dose used).	P10L13-17<br>Suppl_Data: Table1
Experimental animals	8	a. Provide details of the animals used, including species, strain, sex, developmental stage (e.g. mean or median age plus age range) and weight (e.g. mean or median weight plus weight range).<br>b. Provide further relevant information such as the source of animals, international strain nomenclature, genetic modification status (e.g. knock-out or transgenic), genotype, health/immune status, drug or test naïve, previous procedures, etc.	P10L8-9

Housing and husbandry	9	Provide details of:<br>a. Housing (type of facility e.g. specific pathogen free [SPF]; type of cage or housing; bedding material; number of cage companions; tank shape and material etc. for fish).<br>b. Husbandry conditions (e.g. breeding programme, light/dark cycle, temperature, quality of water etc for fish, type of food, access to food and water, environmental enrichment).<br>c. Welfare-related assessments and interventions that were carried out prior to, during, or after the experiment.	P10L4-7
Sample size	10	a. Specify the total number of animals used in each experiment, and the number of animals in each experimental group.<br>b. Explain how the number of animals was arrived at. Provide details of any sample size calculation used.<br>c. Indicate the number of independent replications of each experiment, if relevant.	P10L7-13
Allocating animals to experimental groups	11	a. Give full details of how animals were allocated to experimental groups, including randomisation or matching if done.<br>b. Describe the order in which the animals in the different experimental groups were treated and assessed.	P11L6-8
Experimental outcomes	12	Clearly define the primary and secondary experimental outcomes assessed (e.g. cell death, molecular markers, behavioural changes).	P5L5-P6-L7
Statistical methods	13	a. Provide details of the statistical methods used for each analysis.<br>b. Specify the unit of analysis for each dataset (e.g. single animal, group of animals, single neuron).<br>c. Describe any methods used to assess whether the data met the assumptions of the statistical approach.	P13L1-3
**RESULTS**			
Baseline data	14	For each experimental group, report relevant characteristics and health status of animals (e.g. weight, microbiological status, and drug or test naïve) prior to treatment or testing. (This information can often be tabulated).	Suppl_Data: Table 1
Numbers analysed	15	a. Report the number of animals in each group included in each analysis. Report absolute numbers (e.g. 10/20, not 50%[2]).<br>b. If any animals or data were not included in the analysis, explain why.	Suppl_Data: Table 1
Outcomes and estimation	16	Report the results for each analysis carried out, with a measure of precision (e.g. standard error or confidence interval).	Table1/Figures
Adverse events	17	a. Give details of all important adverse events in each experimental group.<br>b. Describe any modifications to the experimental protocols made to reduce adverse events.	No adverse events
**DISCUSSION**			
Interpretation/ scientific implications	18	a. Interpret the results, taking into account the study objectives and hypotheses, current theory and other relevant studies in the literature.<br>b. Comment on the study limitations including any potential sources of bias, any limitations of the animal model, and the imprecision associated with the results[2].<br>c. Describe any implications of your experimental methods or findings for the replacement, refinement or reduction (the 3Rs) of the use of animals in research.	P6L10-P7L2<br>P7L3-12
Generalisability/ translation	19	Comment on whether, and how, the findings of this study are likely to translate to other species or systems, including any relevance to human biology.	P9L16-P10L1
Funding	20	List all funding sources (including grant number) and the role of the funder(s) in the study.	'Acknowledgments'

NC3Rs

References:
1. Kilkenny C, Browne WJ, Cuthill IC, Emerson M, Altman DG (2010) Improving Bioscience Research Reporting: The ARRIVE Guidelines for Reporting Animal Research. *PLoS Biol* 8(6): e1000412. doi:10.1371/journal.pbio.1000412
2. Schulz KF, Altman DG, Moher D, the CONSORT Group (2010) CONSORT 2010 Statement: updated guidelines for reporting parallel group randomised trials. *BMJ* 340:c332.

Figure 1

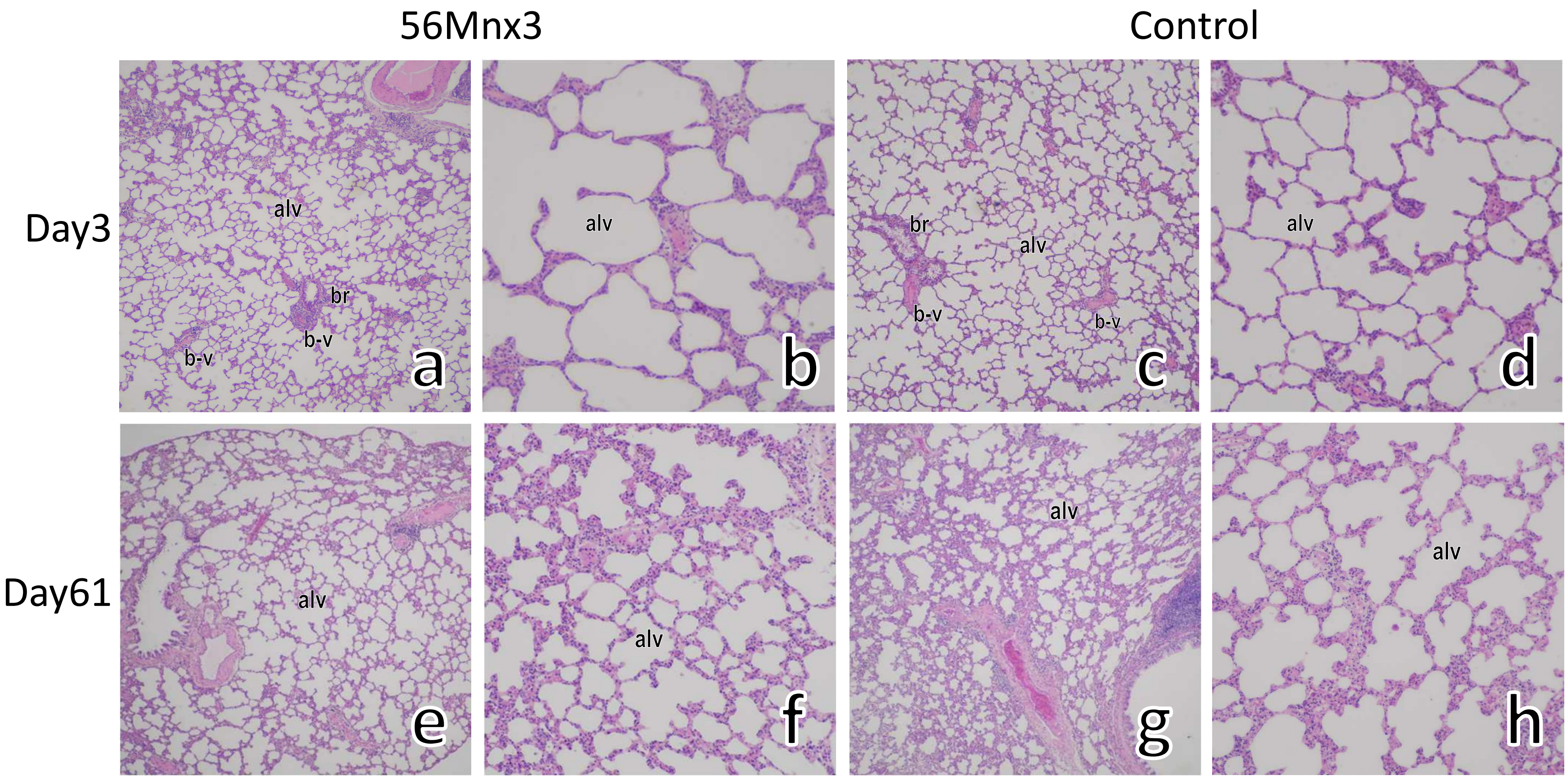
56Mnx3
Control
Day3
Day61
alv
br
b-v
b-v
a
alv
b
br
alv
b-v
b-v
c
alv
d
alv
e
alv
f
alv
g
alv
h

Figure 2

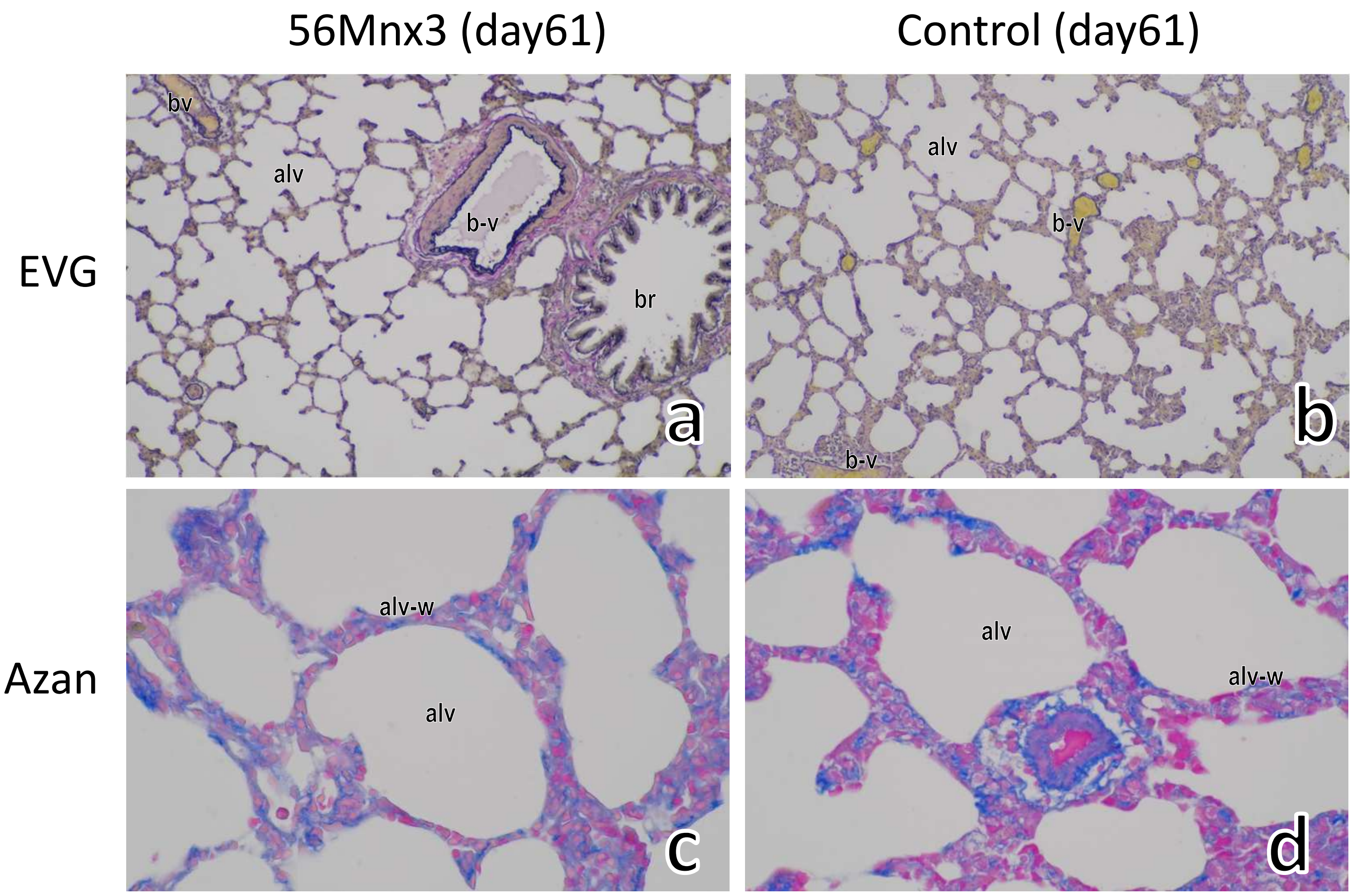

Table 1

	Day3					
	Rat	BW	Necropsy	B.W.	lung	lung
	Stork #	pre-exposr	date	at necrops	(g)	(g/kg bw)
		14Sep2018		20-22Sep2018		
Mn56x3	1-01	184	21Sep2018	179	1.11	6.2
	1-02	216	21Sep2018	221	1.23	5.6
	1-03	220	21Sep2018	231	1.35	5.8
	1-04	255	21Sep2018	248	1.52	6.1
	1-05	259	21Sep2018	259	1.46	5.6
	1-06	281	21Sep2018	278	1.64	5.9
	1-07	244	21Sep2018	245	1.15	4.7
	Mean	237		237	1.35	5.7
	S.E.	12		12	0.08	0.2
Mn56x2	2-01	198	21Sep2018	191	1.25	6.5
	2-02	213	21Sep2018	206	1.34	6.5
	2-03	223	21Sep2018	222	1.95	8.8
	2-04	251	21Sep2018	253	1.19	4.7
	2-05	259	21Sep2018	257	1.50	5.8
	2-06	294	21Sep2018	285	1.51	5.3
	2-07	312	21Sep2018	304	1.92	6.3
	Mean	250		245	1.52	6.3
	S.E.	16		16	0.12	0.5
Mn56x1	3-01	207	20Sep2018	193	1.00	5.2
	3-02	220	20Sep2018	205	1.51	7.4
	3-03	245	20Sep2018	223	1.53	6.9
	3-04	263	20Sep2018	252	1.17	4.6
	3-05	264	20Sep2018	250	1.26	5.0
	3-06	279	20Sep2018	253	1.28	5.1
	3-07	326	20Sep2018	268	1.27	4.7
	Mean	258		235	1.29	5.6
	S.E.	15		11	0.07	0.4
Co-60	4-01	179	22Sep2018	178	1.08	6.1
	4-02	211	22Sep2018	217	1.12	5.2
	4-03	219	22Sep2018	216	1.19	5.5
	4-04	245	22Sep2018	228	1.60	7.0
	4-05	245	22Sep2018	252	1.64	6.5
	4-06	268	22Sep2018	247	1.14	4.6
	4-07	289	22Sep2018	300	1.29	4.3
	Mean	237		234	1.29	5.6
	S.E.	14		14	0.09	0.4
Cold Mn	5-01	185	20Sep2018	175	1.57	9.0
	5-02	206	20Sep2018	194	1.05	5.4
	5-03	229	20Sep2018	227	1.23	5.4
	5-04	248	20Sep2018	245	2.03	8.3
	5-05	268	20Sep2018	261	1.38	5.3
	5-06	276	20Sep2018	265	1.49	5.6
	5-07	301	20Sep2018	276	1.48	5.4
	Mean	245		235	1.46	6.3
	S.E.	15		14	0.12	0.6
Control	6-01	179	22Sep2018	180	0.94	5.2
	6-02	214	22Sep2018	221	1.21	5.5
	6-03	227	22Sep2018	230	1.47	6.4
	6-04	248	22Sep2018	247	2.13	8.6
	6-05	264	22Sep2018	268	1.26	4.7
	6-06	268	22Sep2018	281	1.87	6.7
	6-07	303	22Sep2018	308	1.59	5.2
	Mean	243		248	1.50	6.0
	S.E.	15		16	0.15	0.5

		Day61				
	Rat	BW	Necropsy	B.W.	Lung	Lung
	Stork #	pre-exposr	date	at necrops	(g)	(g/kg bw)
		14Sep201	18-20Sep2018			
	1-08	213	Dead on 20-Oct			
	1-09	217	19Nov2018	313	1.51	4.8
	1-10	244	19Nov2018	314	2.17	6.9
	1-11	244	19Nov2018	329	1.59	4.8
	1-12	278	19Nov2018	360	1.77	4.9
	1-13	291	19Nov2018	415	2.02	4.9
	1-14	311	19Nov2018	389	2.00	5.1
	Mean	257		353	1.84	5.2
	S.E.	14		17	0.11	0.3
	2-08	207	19Nov2018	272	1.46	5.4
	2-09	218	19Nov2018	312	2.54	8.1
	2-10	227	19Nov2018	301	2.20	7.3
	2-11	259	19Nov2018	362	1.98	5.5
	2-12	273	19Nov2018	367	2.09	5.7
	2-13	299	19Nov2018	336	1.76	5.2
	2-14	313	19Nov2018	407	1.89	4.6
	Mean	257		337	1.99	6.0
	S.E.	15		17	0.13	0.5
	3-08	215	18Nov2018	294	1.68	5.7
	3-09	234	18Nov2018	338	1.40	4.1
	3-10	256	18Nov2018	323	1.33	4.1
	3-11	263	18Nov2018	372	2.03	5.5
	3-12	271	18Nov2018	402	2.18	5.4
	3-13	303	18Nov2018	420	1.91	4.5
	3-14	291	18Nov2018	450	2.18	4.8
	Mean	262		371	1.82	4.9
	S.E.	12		21	0.13	0.2
	4-08	195	20Nov2018	229	1.94	8.5
	4-09	214	20Nov2018	309	1.62	5.2
	4-10	233	20Nov2018	296	1.84	6.2
	4-11	228	20Nov2018	312	2.59	8.3
	4-12	253	20Nov2018	358	2.03	5.7
	4-13	278	20Nov2018	406	2.25	5.5
	4-14	306	20Nov2018	386	2.01	5.2
	Mean	244		328	2.04	6.4
	S.E.	14		23	0.12	0.5
	5-08	198	18Nov2018	264	1.69	6.4
	5-09	207	18Nov2018	307		
	5-10	234	18Nov2018	325	1.76	5.4
	5-11	254	18Nov2018	318	1.77	5.6
	5-12	270	18Nov2018	363	2.01	5.5
	5-13	294	18Nov2018	372	1.67	4.5
	5-14	319	18Nov2018	413	1.79	4.3
	Mean	254		337	1.78	5.3
	S.E.	17		19	0.05	0.3
208	6-08	208	20Nov2018	260	1.98	7.6
227	6-09	227	20Nov2018	306	2.53	8.3
228	6-10	228	20Nov2018	290	1.93	6.7
273	6-11	273	20Nov2018	367	2.48	6.8
267	6-12	267	20Nov2018	371	1.73	4.7
283	6-13	283	20Nov2018	356	1.77	5.0
309	6-14	309	20Nov2018	359	2.31	6.4
	Mean	256		330	2.10	6.5
	S.E.	14		17	0.13	0.5

		B.W.	Lung	Lung/bw
	Dunnet test	p.value.ad	p.value.adj	p.value.adj
Mn56x3 vs C	1 vs 6	0.967	0.780	0.941
Mn56x2 vs C	2 vs 6	1.000	1.000	0.993
Mn56x1 vs C	3 vs 6	0.919	0.538	0.889
Co60 vs C	4 vs 6	0.932	0.588	0.907
Mn-C vs C	5 vs 6	0.944	1.000	0.990

	B.W.	Lung	Lung/bw
Dunnet test	p.value.adj	p.value.adj	p.value.adj
1 vs 6	0.805	0.394	0.189
2 vs 6	0.999	0.933	0.883
3 vs 6	0.461	0.393	0.063
4 vs 6	1.000	0.992	1.000
5 vs 6	0.998	0.145	0.204

		B.W.	Lung	Lung/bw
	Dunnet test	p.value.ad	p.value.adj	p.value.adj
Mn56x3 vs Mn-C	1 vs 5	1.000	0.864	0.700
Mn56x2 vs Mn-C	2 vs 5	0.978	0.992	1.000
Mn56x1 vs Mn-C	3 vs 5	1.000	0.572	0.645

	B.W.	Lung	Lung/bw
Dunnet test	p.value.adj	p.value.adj	p.value.adj
1 vs 5	0.951	0.985	1.000
2 vs 5	1.000	0.551	0.675
3 vs 5	0.650	1.000	0.804

Figure 3 - Q-PCR results - Col-I, Elast, Tgfb, Smad2 and Smad7 in the lung on postexposure day 3 and day 61

Day3

		b-actin	CoI-1	x1000/bact	Elast	x1000/bact	TGFb	x1000/bact	Smad2	x1000/bact	Smad7	x1000/ba
Mn56x3	1-01	375	265	708	73.5	196	158	421	9.5	25	2.3	6.3
	1-02	172	110	639	28.0	163	55	320	3.9	23	1.1	6.3
	1-03	191	98	514	28.5	149	60	314	4.3	22	1.2	6.1
	1-04	379	408	1075	45.4	120	116	307	10.5	28	2.6	6.8
	1-05	618	614	993	46.8	76	177	286	17.4	28	3.5	5.7
	1-06	519	495	954	47.6	92	175	337	14.5	28	3.0	5.7
	1-07	152	79	518	6.3	41	29	189	3.1	20	0.8	5.0
				772		120		310		25		6.0
				88		20		26		1.2		0.22
Mn56x2	2-01	293	237	808	20.8	71	109	372	8.0	27	1.3	4.4
	2-02	412	260	632	41.6	101	237	575	13.4	32	1.9	4.6
	2-03	374	231	617	23.4	63	143	382	11.7	31	2.0	5.4
	2-04	453	394	869	33.1	73	175	385	12.3	27	2.4	5.2
	2-05	406	151	372	15.5	38	161	395	8.6	21	2.0	4.9
	2-06	564	697	1235	41.1	73	180	319	16.2	29	3.7	6.6
	2-07	324	293	903	45.0	139	113	349	8.6	26	2.1	6.4
				777		80		397		28		5.4
				103		12		31		1.4		0.32
Mn56x1	3-01	242	180	745	19.6	81	86	357	7.0	29	1.7	7.2
	3-02	294	97	331	21.2	72	102	348	5.1	17	1.2	4.2
	3-03	312	281	901	34.3	110	140	449	6.9	22	1.8	5.9
	3-04	299	212	707	26.5	89	88	294	5.6	19	1.3	4.3
	3-05	234	109	464	17.2	74	74	315	4.4	19	0.9	3.8
	3-06	203	126	621	17.5	86	56	277	4.7	23	1.1	5.4
	3-07	254	239	940	20.7	81	82	322	5.8	23	1.3	4.9
				673		85		337		22		5.1
				84		5		21		1.5		0.44
60Co	4-01	284	208	734	29.4	103	108	380	7.2	25	1.3	4.5
	4-02	310	282	911	25.6	83	66	213	7.2	23	1.2	3.9
	4-03	206	112	544	28.0	136	47	230	3.6	17	0.7	3.3
	4-04	289	137	475	25.9	90	69	238	4.4	15	1.1	3.7
	4-05	298	201	676	24.5	82	109	367	5.8	20	1.4	4.6
	4-06	307	914	2979	43.5	142	94	307	8.6	28	2.2	7.0
	4-07	169	257	1523	20.6	122	49	291	4.7	28	0.9	5.3
				1120		108		290		22		4.6
				337		9		25		1.9		0.48
Cold Mn	5-01	351	429	1220	56.6	161	131	373	9.9	28	1.8	5.3
	5-02	289	254	879	43.3	150	132	456	7.7	27	1.6	5.4
	5-03	276	244	885	55.1	199	79	287	6.2	22	1.4	5.2
	5-04	260	172	659	35.6	137	95	365	5.5	21	1.2	4.5
	5-05	241	129	537	25.5	106	93	386	7.2	30	1.2	4.9
	5-06	287	249	867	34.5	120	91	316	6.3	22	1.2	4.2
	5-07	270	126	466	23.9	89	121	446	7.1	26	0.7	2.6
				787		137		376		25		4.6
				97		14		23		1.3		0.37
Control	6-01	455	391	860	56.8	125	139	305	23.7	52	2.1	4.7
	6-02	585	457	781	89.7	153	111	189	17.1	29	3.3	5.6
	6-03	310	295	952	58.1	187	86	277	9.7	31	1.2	3.9
	6-04	405	210	517	40.4	100	119	295	11.3	28	1.8	4.5
	6-05	89	62	703	12.1	136	71	805	1.9	22	0.3	3.1
	6-06	398	324	812	34.7	87	82	205	8.2	21	2.3	5.8
	6-07	336	235	699	32.2	96	67	200	9.1	27	1.5	4.3
				761		126		325		30		4.6
				52		14		82		3.9		0.35

Day61

		b-actin	CoI-1	x1000/bact	Elast	x1000/bact	TGFb	x1000/bact	Smad2	x1000/bact	Smad7	x1000/ba
Mn56x3	1-09	322	421	1304	102.0	316	145	451	10.7	33	1.4	4.3
	1-10	441	277	627	86.1	195	252	572	10.1	23	2.3	5.1
	1-11	304	221	727	92.4	303	86	282	8.4	28	1.8	5.8
	1-12	286	209	732	47.4	166	107	375	8.0	28	1.4	5.0

	1-13	297	232	782	51.8	175	77	260	7.2	24	1.6	5.3
	1-14	507	298	587	47.0	93	75	148	7.1	14	2.8	5.5
				793		208		348		25		5.2
				106		35		62		2.6		0.20
Mn56x2	2-08	170	68	402	20.7	122	35	205	2.8	16	0.7	4.2
	2-09	366	176	479	32.6	89	165	451	7.7	21	1.7	4.6
	2-10	296	89	301	17.4	59	97	326	5.3	18	1.3	4.3
	2-11	205	166	807	22.4	109	56	271	5.1	25	0.7	3.3
	2-12	234	109	466	21.0	90	66	281	5.9	25	0.9	3.9
	2-13	225	67	298	18.7	83	60	268	4.9	22	1.2	5.3
	2-14	263	140	533	26.6	101	67	255	7.4	28	1.2	4.7
				470		93		294		22		4.3
				66		8		29		1.6		0.24
Mn56x1	3-08	178	117	655	32.7	184	56	314	4.0	22	0.9	5.3
	3-09	173	84	484	20.6	119	50	287	4.0	23	0.8	4.9
	3-10	89	35	388	8.9	99	21	236	1.5	16	0.3	3.2
	3-11	246	189	768	33.0	134	88	356	4.8	20	1.1	4.5
	3-12	329	150	456	19.5	59	69	210	4.0	12	1.1	3.3
	3-13	277	370	1339	99.8	361	67	244	5.2	19	1.3	4.6
	3-14	242	221	916	31.7	131	51	210	4.2	17	0.8	3.2
				715		155		265		19		4.1
				126		37		21		1.4		0.33
60Co	4-08	366	228	622	55.9	153	142	387	10.1	28	1.8	5.0
	4-09	358	334	933	78.6	220	107	299	8.8	25	1.7	4.7
	4-10	355	210	592	74.3	210	120	338	11.2	32	2.2	6.1
	4-11	492	285	578	61.7	125	191	389	11.0	22	1.8	3.6
	4-12	372	241	647	69.9	188	131	352	7.9	21	1.8	4.9
	4-13	302	165	547	31.4	104	84	279	6.1	20	1.3	4.1
	4-14	166	114	687	27.4	165	36	219	3.4	20	0.7	4.2
				658		166		323		24		4.7
				49		16		23		1.6		0.31
Cold Mn	5-08	217	93	429	13.2	61	61	282	4.1	19	0.9	4.1
	5-09	422	281	667	32.2	76	100	237	10.2	24	2.2	5.1
	5-10	368	229	622	28.7	78	82	224	8.1	22	1.8	4.8
	5-11	467	288	616	29.1	62	84	179	10.5	23	2.4	5.1
	5-12	425	322	757	23.2	55	79	185	9.0	21	2.6	6.1
	5-13	321	154	480	24.2	75	57	178	7.8	24	1.6	4.9
	5-14	362	314	867	27.5	76	87	241	9.4	26	2.4	6.5
				634		69		218		23		5.2
				57		4		15		0.9		0.31
Control	6-08	405	225	555	55.3	137	139	342	10.5	26	1.9	4.7
	6-09	399	274	687	54.3	136	102	255	9.9	25	1.9	4.8
	6-10	354	167	471	30.3	86	116	328	11.2	32	2.2	6.2
	6-11	228	141	618	16.8	73	38	167	2.6	11	1.0	4.2
	6-12	335	381	1137	44.3	132	88	262	8.7	26	1.7	5.1
	6-13	279	228	815	42.1	151	80	288	9.8	35	1.9	6.7
	6-14	424	243	573	33.5	79	124	294	9.9	23	2.1	5.0
				694		113		276		25		5.2
				85		12		22		2.8		0.34

Day3	Dunnet test	p.value.adj	p.value.adj	p.value.adj	p.value.adj	p.value.adj
Mn56x3 vs C	1 vs 6	1.00	1.00	1.00	0.45	0.02
Mn56x2 vs C	2 vs 6	1.00	0.09	0.66	0.89	0.33
Mn56x1 vs C	3 vs 6	0.88	0.07	1.00	0.17	0.80
Co60 vs C	4 vs 6	0.80	0.69	0.92	0.23	1.00
Mn-C vs C	5 vs 6	1.00	0.96	0.81	0.49	1.00
Mn56x3 vs Mn-C	1 vs 5	1.00	0.90	0.31	1.00	0.04
Mn56x2 vs Mn-C	2 vs 5	1.00	0.03	0.98	0.60	0.39
Mn56x1 vs Mn-C	3 vs 5	0.84	0.03	0.68	0.34	0.83
Day61						
Mn56x3 vs C	1 vs 6	0.90	0.15	0.74	0.45	1.00
Mn56x2 vs C	2 vs 6	0.19	0.55	0.99	0.89	0.16
Mn56x1 vs C	3 vs 6	1.00	0.75	1.00	0.17	0.13

Co60 vs C	4 vs 6	0.99	0.09	0.50	0.23	0.57
Mn-C vs C	5 vs 6	0.96	0.05	0.18	0.49	1.00
Mn56x3 vs Mn-C	1 vs 5	0.62	0.05	0.15	0.31	1.00
Mn56x2 vs Mn-C	2 vs 5	0.30	0.08	0.55	0.19	0.14
Mn56x1 vs Mn-C	3 vs 5	0.97	0.22	0.75	0.35	0.12

Figure 4- Q-PCR results - AQP1, AQP4 and AQP5 in the lung on postexposure day 3 and day 61

Day3

		b-actin	AQP1	x1000/bact	AQP4	x1000/bact	AQP5	x1000/bact
Mn56x3	1-01	375	25.1	66.9	3.3	8.8	5.5	14.5
	1-02	172	10.3	59.8	2.9	17.0	2.7	15.7
	1-03	191	10.2	53.5	1.6	8.3	2.9	15.2
	1-04	379	16.1	42.5	4.4	11.7	6.0	15.8
	1-05	618	26.6	42.9	8.2	13.3	8.9	14.3
	1-06	519	20.9	40.3	4.6	8.9	7.1	13.8
	1-07	152	4.5	29.9	1.4	9.0	-	
				48.0		11.0		14.9
				4.8		1.2		0.3
Mn56x2	2-01	293	18.0	61.5	3.8	13.1	4.0	13.7
	2-02	412	19.0	46.2	3.6	8.6	5.7	13.9
	2-03	374	18.8	50.2	4.0	10.8	4.8	12.9
	2-04	453	26.4	58.3	4.3	9.5	8.9	19.6
	2-05	406	15.2	37.5	5.4	13.4	5.9	14.5
	2-06	564	22.3	39.6	11.0	19.5	8.9	15.8
	2-07	324	19.4	60.0	3.2	9.8	5.7	17.5
				50.5		12.1		15.4
				3.7		1.4		0.9
Mn56x1	3-01	242	21.7	90.0	2.1	8.8	2.8	11.4
	3-02	294	8.8	29.8	2.0	6.8	1.5	4.9
	3-03	312	12.6	40.3	3.0	9.5	2.8	9.1
	3-04	299	9.8	32.8	1.9	6.3	2.0	6.8
	3-05	234	11.1	47.5	2.4	10.2	1.4	6.1
	3-06	203	14.3	70.4	2.7	13.1	1.8	8.7
	3-07	254	17.5	68.8	5.1	20.1	3.3	13.0
				54.2		10.7		8.6
				8.5		1.8		1.1
60Co	4-01	284	17.2	60.4	3.1	11.1	2.3	8.1
	4-02	310	20.4	65.8	4.1	13.1	3.7	11.9
	4-03	206	11.8	57.1	1.9	9.2	1.5	7.2
	4-04	289	9.3	32.1	1.7	6.0	1.2	4.2
	4-05	298	8.9	30.0	1.9	6.3	2.2	7.5
	4-06	307	26.1	85.0	7.9	25.6	4.5	14.6
	4-07	169	13.4	79.2	2.9	17.3	2.1	12.4
				58.5		12.7		9.4
				8.0		2.6		1.4
Cold Mn	5-01	351	17.7	50.4	6.1	17.3	3.8	10.7
	5-02	289	20.4	70.6	4.1	14.0	3.3	11.3
	5-03	276	15.7	57.0	2.9	10.5	2.9	10.7
	5-04	260	14.4	55.4	2.9	11.3	2.4	9.2
	5-05	241	21.4	88.7	2.7	11.2	2.5	10.4
	5-06	287	14.5	50.5	2.3	7.9	3.1	10.7
	5-07	270	16.3	60.4	1.4	5.1	1.7	6.2
				61.8		11.0		9.9
				5.2		1.5		0.7
Control	6-01	455	22.2	48.8	4.8	10.5	3.6	7.8
	6-02	585	27.5	47.0	9.4	16.1	3.8	6.5
	6-03	310	21.6	69.7	2.7	8.8	3.7	11.9
	6-04	405	20.2	50.0	4.7	11.6	1.9	4.6
	6-05	89	4.8	53.7	2.7	30.6	1.1	12.8
	6-06	398	15.9	39.9	1.6	4.1	6.5	16.4
	6-07	336	-		1.7	5.0	2.3	6.8
				51.5		12.4		9.6
				4.1		3.4		1.6

Day61

		b-actin	AQP1	x1000/bact	AQP4	x1000/bact	AQP5	x1000/bact
Mn56x3	1-09	322	19.4	60.2	5.9	18.4	5.9	18.4
	1-10	441	23.7	53.6	4.8	10.8	5.4	12.3
	1-11	304	21.2	69.6	4.8	15.7	3.9	12.7
	1-12	286	14.1	49.3	3.4	12.0	3.3	11.4
	1-13	297	18.2	61.4	4.1	13.8	3.1	10.5
	1-14	507	14.8	29.2	2.8	5.5	4.0	7.9
				53.9		12.7		12.2
				5.7		1.8		1.4
Mn56x2	2-08	170	10.9	64.0	0.9	5.4	1.5	8.7

	2-09	366	10.7	29.1	2.4	6.7	2.6	7.0
	2-10	296	7.9	26.5	2.2	7.4	1.7	5.7
	2-11	205	12.4	60.6	4.4	21.2	2.1	10.2
	2-12	234	12.5	53.3	2.7	11.7	2.2	9.3
	2-13	225	13.7	61.0	1.9	8.2	2.8	12.5
	2-14	263	14.1	53.5	1.8	7.0	3.4	12.8
				49.7		9.7		9.5
				5.9		2.1		1.0
Mn56x1	3-08	178	11.8	66.2	2.3	13.0	2.0	11.2
	3-09	173	8.4	48.9	1.5	8.9	1.4	8.1
	3-10	89	2.9	32.5	0.6	6.6	0.8	8.8
	3-11	246	8.2	33.4	1.8	7.4	1.9	7.6
	3-12	329	8.1	24.6	1.5	4.5	1.5	4.6
	3-13	277	11.7	42.1	3.9	14.2	2.2	8.1
	3-14	242	12.9	53.3	1.4	5.7	1.9	8.0
				43.0		8.6		8.0
				5.4		1.4		0.7
60Co	4-08	366	15.2	41.4	5.7	15.7	3.7	10.0
	4-09	358	17.5	48.8	7.7	21.4	4.1	11.3
	4-10	355	15.8	44.6	9.6	27.0	4.0	11.3
	4-11	492	12.1	24.5	5.5	11.1	3.0	6.1
	4-12	372	15.5	41.6	4.2	11.4	3.6	9.7
	4-13	302	9.4	31.1	1.8	6.1	2.0	6.5
	4-14	166	7.4	44.5	3.8	23.0	1.6	9.9
				39.5		16.5		9.3
				3.2		2.9		0.8
Cold Mn	5-08	217	5.2	24.0	2.4	11.1	2.2	10.0
	5-09	422	16.9	40.0	6.6	15.6	5.0	11.8
	5-10	368	10.7	29.1	2.4	6.4	2.7	7.4
	5-11	467	17.6	37.8	2.8	6.1	5.2	11.1
	5-12	425	14.6	34.4	1.9	4.5	5.0	11.8
	5-13	321	14.9	46.3	3.2	9.8	4.2	13.0
	5-14	362	17.7	48.9	4.9	13.6	4.6	12.7
				37.2		9.6		11.1
				3.4		1.6		0.7
Control	6-08	405	15.2	37.5	4.3	10.5	3.2	7.8
	6-09	399	12.1	30.4	4.6	11.4	2.6	6.5
	6-10	354	17.3	48.9	4.1	11.7	4.2	11.9
	6-11	228	5.4	23.8	2.0	8.8	1.1	4.6
	6-12	335	19.3	57.4	6.6	19.6	4.3	12.8
	6-13	279	18.6	66.7	5.0	18.0	4.6	16.4
	6-14	424	14.8	34.9	4.8	11.4	2.9	6.8
				42.8		13.1		9.6
				5.8		1.5		1.6

Day3		Dunnet test	p.value.adj	p.value.adj	p.value.adj
	Mn56x3 vs C	1 vs 6	0.98	0.97	0.04
	Mn56x2 vs C	2 vs 6	1.00	1.00	0.03
	Mn56x1 vs C	3 vs 6	1.00	0.96	0.94
	Co60 vs C	4 vs 6	0.91	1.00	1.00
	Mn-C vs C	5 vs 6	0.47	0.98	1.00
	Mn56x3 vs Mn-C	1 vs 5	0.25	1.00	0.00
	Mn56x2 vs Mn-C	2 vs 5	0.33	0.98	0.00
	Mn56x1 vs Mn-C	3 vs 5	0.90	1.00	0.80
Day61					
	Mn56x3 vs C	1 vs 6	0.51	1.00	0.51
	Mn56x2 vs C	2 vs 6	0.83	0.60	1.00
	Mn56x1 vs C	3 vs 6	1.00	0.20	0.77
	Co60 vs C	4 vs 6	0.97	0.76	1.00
	Mn-C vs C	5 vs 6	0.84	0.44	0.74
	Mn56x3 vs Mn-C	1 vs 5	0.14	0.61	0.95
	Mn56x2 vs Mn-C	2 vs 5	0.33	1.00	0.60
	Mn56x1 vs Mn-C	3 vs 5	0.86	0.99	0.05

Figure 5 - AQP5 original scan

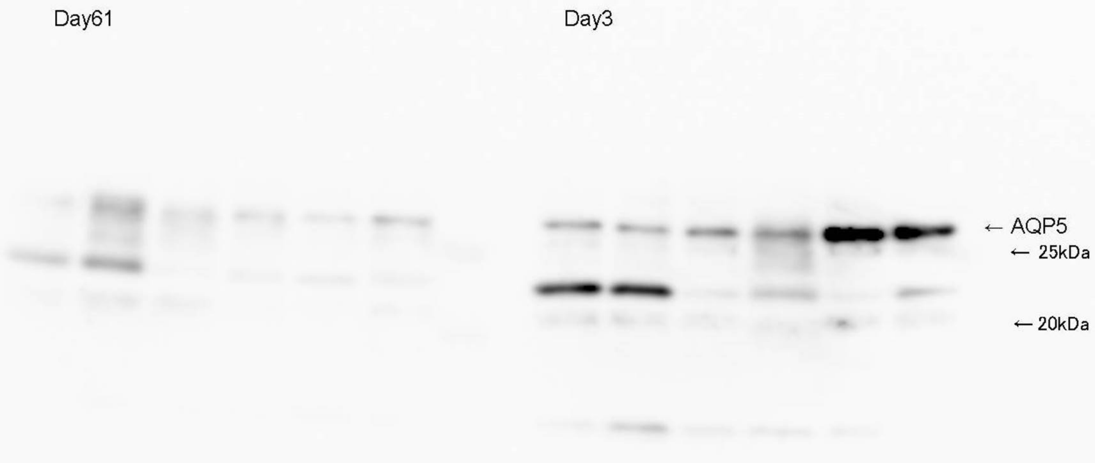

Figure 5 - betaActin original scan

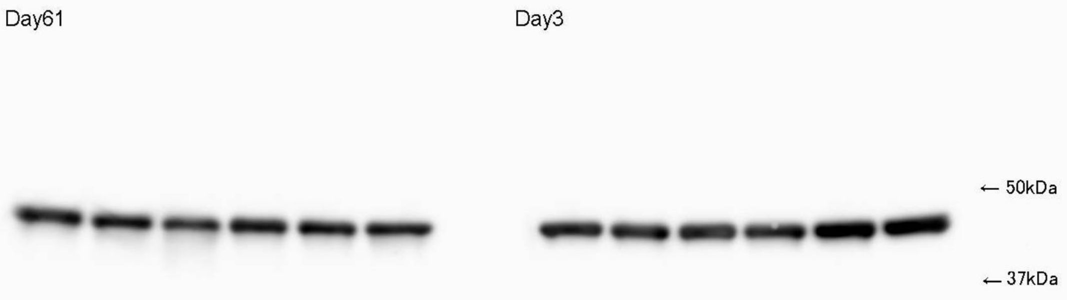

Figure 5 – filter photo

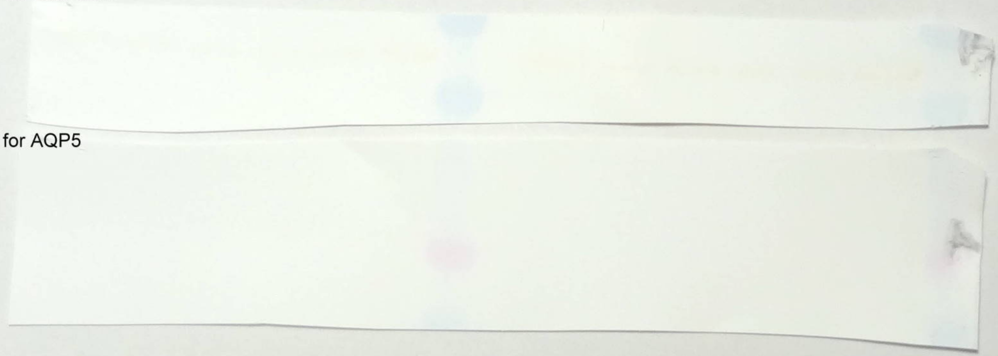

Figure 5 - Western Blot Densitometry

Day3				Day61		
		AQP5/Bact				AQP5/Bact
Mn56x3	1-01	2.98		Mn56x3	1-09	1.05
	1-02	3.07			1-10	1.29
	1-03	2.63			1-11	0.70
	1-04	2.97			1-14	0.81
		2.91	0.10			
Mn56x2	2-01	2.66		Mn56x2	2-08	0.72
	2-02	1.83			2-09	1.55
	2-03	2.86			2-10	1.31
	2-04	2.88			2-14	1.44
		2.56	0.25			
Mn56x1	3-01	1.76		Mn56x1	3-08	0.80
	3-02	2.24			3-10	0.86
	3-03	1.63			3-11	1.17
	3-04	1.85			3-14	0.69
		1.87	0.13			
60Co	4-01	1.17		60Co	4-08	1.40
	4-02	1.08			4-09	1.64
	4-03	2.01			4-10	1.16
	4-04	1.55			4-14	0.74
		1.45	0.21			
Cold Mn	5-01	0.92		Cold Mn	5-08	2.25
	5-02	1.38			5-09	1.03
	5-03	0.98			5-10	0.93
	5-04	1.02			5-14	0.76
		1.07	0.10			
Control	6-01	0.92		Control	6-08	1.33
	6-02	0.82			6-09	1.00
	6-03	1.11			6-10	0.90
	6-04	1.15			6-14	0.67
		1.00	0.08			

Day3		
	Mn56x3 vs C	0.000
	Mn56x2 vs C	0.030
	Mn56x1 vs C	0.017
	Co60 vs C	0.384
	Mn-C vs C	0.973
	Mn56x3 vs Mn-C	0.000
	Mn56x2 vs Mn-C	0.018
	Mn56x1 vs Mn-C	0.019

Day61		
	Mn56x3 vs C	1.000
	Mn56x2 vs C	0.680
	Mn56x1 vs C	0.971
	Co60 vs C	0.743
	Mn-C vs C	0.917
	Mn56x3 vs Mn-C	0.774
	Mn56x2 vs Mn-C	1.000
	Mn56x1 vs Mn-C	0.632
